# Proteolytic activation of the epithelial sodium channel (ENaC) by the cysteine protease cathepsin-S

**DOI:** 10.1007/s00424-012-1138-3

**Published:** 2012-08-05

**Authors:** Silke Haerteis, Matteus Krappitz, Marko Bertog, Annabel Krappitz, Vera Baraznenok, Ian Henderson, Erik Lindström, Jane E. Murphy, Nigel W. Bunnett, Christoph Korbmacher

**Affiliations:** 1 Institut für Zelluläre und Molekulare Physiologie, Friedrich-Alexander-Universität Erlangen-Nürnberg, Waldstr. 6, 91054 Erlangen, Germany; 2 Medivir AB, Huddinge, Sweden; 3 Center for the Neurobiology of Digestive Diseases, Department of Surgery, University of California San Francisco, San Francisco, CA USA; 4 Monash Institute of Pharmaceutical Sciences, 381 Royal Parade, Parkville, VIC 3052 Australia

**Keywords:** Epithelial sodium channel (ENaC), Proteolytic channel activation, Cathepsin, Two-electrode voltage clamp, Transepithelial Na^+^ transport

## Abstract

Proteolytic processing of the amiloride-sensitive epithelial sodium channel (ENaC) by serine proteases is known to be important for channel activation. Inappropriate ENaC activation by proteases may contribute to the pathophysiology of cystic fibrosis and could be involved in sodium retention and the pathogenesis of arterial hypertension in the context of renal disease. We hypothesized that in addition to serine proteases, cathepsin proteases may activate ENaC. Cathepsin proteases belong to the group of cysteine proteases and play a pathophysiological role in inflammatory diseases. Under pathophysiological conditions, cathepsin-S (Cat-S) may reach ENaC in the apical membrane of epithelial cells. The aim of this study was to investigate the effect of purified Cat-S on human ENaC heterologously expressed in *Xenopus laevis* oocytes and on ENaC-mediated sodium transport in cultured M-1 mouse renal collecting duct cells. We demonstrated that Cat-S activates amiloride-sensitive whole-cell currents in ENaC-expressing oocytes. The stimulatory effect of Cat-S was preserved at pH 5. ENaC stimulation by Cat-S was associated with the appearance of a γENaC cleavage fragment at the plasma membrane indicating proteolytic channel activation. Mutating two valine residues (V182 and V193) in the critical region of γENaC prevented proteolytic activation of ENaC by Cat-S. Pre-incubation of the oocytes with the Cat-S inhibitor morpholinurea-leucine-homophenylalanine-vinylsulfone-phenyl (LHVS) prevented the stimulatory effect of Cat-S on ENaC. In contrast, LHVS had no effect on ENaC activation by the prototypical serine proteases trypsin and chymotrypsin. Cat-S also stimulated ENaC in differentiated renal epithelial cells. These findings demonstrate that the cysteine protease Cat-S can activate ENaC which may be relevant under pathophysiological conditions.

## Introduction

The epithelial sodium channel (ENaC) is localized in the apical membrane of the aldosterone-sensitive distal nephron, distal colon, respiratory epithelia, and ducts of salivary and sweat glands. In these epithelia, ENaC is the rate-limiting transport mechanism for sodium absorption.

ENaC is a member of the ENaC/degenerin family of non-voltage-gated ion channels which also includes the acid-sensing ion channel ASIC1. The available crystal structure of chicken ASIC1 [9, 29, 50] and recent atomic force microscopy data of ENaC [49] suggest that ENaC is a heterotrimer composed of three homologous subunits α, β, and γ. Each subunit of ENaC contains two transmembrane domains, a large extracellular domain, and short intracellular amino and carboxyl termini. In humans, an additional δ-subunit exists which can functionally replace the α-subunit in heterologous expression systems [20, 30, 54, 56].

A unique feature of ENaC regulation is its proteolytic processing thought to be critical for channel activation under (patho-)physiological conditions [32, 47]. However, the precise molecular mechanisms of proteolytic channel activation remain a matter of debate. The channel is thought to be in its mature and active form in its cleaved state, but there is evidence for the simultaneous presence of both cleaved and non-cleaved ENaC in the plasma membrane. Proteases activate ENaC by cleaving specific sites in the extracellular domains of the α-, γ-, and δ-subunit but not the β-subunit [1, 17, 20, 28, 44, 47]. Cleavage probably results in the release of inhibitory peptides thereby activating the channel by a change in its conformation [21, 32]. Intracellular proteolytic cleavage by furin [27] at three distinct furin sites (two in the α-subunit and one in the γ-subunit) is thought to be important for ENaC maturation during the biosynthetic pathway before the channel reaches the plasma membrane [32]. The second and final activating cleavage event probably takes place at the plasma membrane where γENaC is cleaved by membrane-bound proteases and/or extracellular proteases in a region distal to the furin site [1, 10, 17, 23]. It has also been reported that proteases may indirectly affect ENaC activity [3, 16]. There is convincing evidence that several serine proteases (e.g., channel-activating proteases (CAP1–3), furin, trypsin, chymotrypsin, plasmin, neutrophil elastase, kallikrein) can proteolytically activate ENaC [47]. In addition to serine proteases, other groups of proteases may be involved in proteolytic ENaC activation. Indeed, recent data demonstrate that co-expression of ENaC and the metalloproteinase meprin β leads to proteolytic activation of rat ENaC [19]. However, at present, the (patho-)physiologically relevant proteases for ENaC activation remain to be determined and may differ from tissue to tissue.

Recently, we and others reported that plasmin can proteolytically activate ENaC [41, 52]. Inappropriate ENaC activation by locally generated proteases may be relevant in several diseases. For example, in the kidney luminal ENaC activation by urinary plasmin — generated from filtered plasminogen which is catalyzed by urokinase-type plasminogen activator — may contribute to renal sodium retention in nephrotic syndrome [52]. Furthermore, enhanced ENaC activity by locally released proteases (e.g., human neutrophil elastase) may aggravate pulmonary symptoms in patients with cystic fibrosis during an inflammatory response to acute respiratory infection [25, 45]. Interestingly, the metalloproteinases meprins are expressed by leukocytes of the intestinal immune system [15]. Thus, ENaC activation by meprin may occur in inflammatory bowel disease. These examples illustrate a possible pathophysiological role of proteolytic ENaC activation in the context of inflammatory diseases. Organ-specific expression of proteases and differences in proteolytic ENaC processing may be responsible for the development of distinct disease phenotypes.

Proteases are classified according to their catalytic active center into six groups: aspartate, glutamic acid, metallo, serine, threonine, and cysteine proteases. Human cysteine proteases such as cathepsins are known to play an important role in a variety of inflammatory/immune diseases and have a wide range of (patho-)physiological effects [5, 37, 46]. In general, cysteine proteases are secreted by macrophages and epithelial cells during injury and disease. Cathepsins, a family of 11 proteases in humans, may play a pathophysiological role in many inflammatory diseases [11, 48]. Under pathophysiological conditions, cathepsin-S (Cat-S) could reach ENaC in the apical membrane of epithelial cells. For example, Cat-S is secreted into the colonic lumen during colitis and may reach ENaC expressed in the apical membrane of colonic epithelial cells [11]. Similarly, ENaC in the distal nephron may be exposed to Cat-S which may be present in the tubular fluid in inflammatory renal disease. The aim of this study was to test the effect of purified Cat-S on human ENaC heterologously expressed in *X. laevis* oocytes and on ENaC-mediated sodium transport in cultured M-1 mouse renal collecting duct cells.

## Material and methods

### Chemicals

Amiloride hydrochloride and α-chymotrypsin from bovine pancreas (type II) were purchased from Sigma. Human neutrophil elastase (hNE) was obtained from Serva Electrophoresis. To prevent contamination of hNE with serine proteases, we always applied hNE in the presence of the serine protease inhibitor aprotinin (Sigma, 10 μM) which does not inhibit neutrophil elastase [1]. Pro-Cat-S was activated by incubation in activation buffer (NaOAc 0.1 M, NaCl 0.1 M, EDTA 5 mM, DTT 1 mM, pH 4.5) at 37 °C for 15 to 30 min (incubation time is batch dependent and determined by measuring the time to peak activity using the assay conditions below). The Cat-S was then buffer-exchanged into PBS (Dulbecco’s phosphate buffered saline pH 7.4, Sigma) using an Econo-Pac 10DG column (Bio-Rad). The active site concentration of Cat-S was determined by titration with E-64 (3-carboxy-trans-2,3-epoxypropyl-leucylamido(4-guanidino)butane) (Sigma) in a buffer of 0.1 M Na phosphate, 0.1 M NaCl, 0.1 % PEG-4000, 1 mM DTT, pH 6.5 using 100 μM boc-Val-Leu-Lys-AMC (Bachem) as substrate and monitoring fluorescence at 390 nm excitation and 460 nm emission. The Cat-S stock solution prepared in PBS was diluted to the working concentration in ND96 solution. An irreversible Cat-S inhibitor (morpholinurea-leucine-homophenylalanine-vinyl phenyl sulfone — LHVS) [2] was used to inhibit the effect of Cat-S on ENaC. Cat-S and LHVS were provided by Medivir AB.

### Peptide

A 23-mer γENaC peptide was synthesized and purified by the Peptide Synthesis Core Facility (University of Calgary, Canada) (purity > 95 %). The peptide sequence (176-TGRKRKVGGSIIHKASNVMHIES-198) corresponds to the amino acid sequence T176 to S198 of the extracellular domain of γENaC thought to be critical for proteolytic channel activation.

### Plasmids

Full-length cDNAs for human wild-type (wt) α-, β-, and γENaC were kindly provided by Harry Cuppens (Leuven, Belgium). They were subcloned into pcDNA3.1 vector, and linearized plasmids were used as templates for cRNA synthesis (mMessage mMachine) using T7 as promoter as described previously [20, 45]. γ_V182G;V193G_ mutant was generated by site-directed mutagenesis (QuikChange® Site-Directed Mutagenesis Kit, Stratagene) and sequences were confirmed (LGC Genomics).

### Isolation of oocytes and injection of cRNA

Oocytes were obtained from adult female *X. laevis* in accordance with the principles of German legislation, with approval by the animal welfare officer for the University of Erlangen-Nürnberg and under the governance of the state veterinary health inspectorate (permit no. 621–2531.32-05/02). Animals were anesthetized in 0.2 % MS222 and ovarian lobes were obtained through a small abdominal incision. After suture, the animals were allowed to recover fully in a separate tank before they were returned to the frog colony 1 day later. Oocytes were isolated from the ovarian lobes by enzymatic digestion at 19 °C for 3–4 h with 600–700 U/ml type 2 collagenase from *Clostridium histolyticum* (CLS 2, Worthington) dissolved in a solution containing (in mM) NaCl 82.5, KCl 2, MgCl_2_ 1, and HEPES 1 (pH 7.4 with NaOH). Defolliculated stage V–VI oocytes were injected (Nanoject II automatic injector, Drummond) with 0.2 ng cRNA per ENaC subunit, unless stated otherwise. The cRNAs were dissolved in RNase-free water and the total volume injected was 46 nl. Injected oocytes were stored at 19 °C in low sodium solution (in mM: *N*-methyl-d-glucamine-Cl 87, NaCl 9, KCl 2, CaCl_2_ 1.8, MgCl_2_ 1, HEPES 5, pH 7.4 with Tris) supplemented with 100 U/ml penicillin and 100 μg/ml streptomycin.

### Two-electrode voltage clamp

Oocytes were routinely studied 2 days after injection using the two-electrode voltage clamp technique essentially as described previously [20, 45]. Individual oocytes were placed in a small experimental chamber and constantly superfused with high sodium solution ND96 (in mM: NaCl 96, KCl 2, CaCl_2_ 1.8, MgCl_2_ 1, HEPES 5, pH 7.4 with Tris) supplemented with amiloride (2 μM) at a rate of 2–3 ml/min at room temperature. For acidic pH, we used a solution containing in mM: NaCl 96, KCl 2, CaCl_2_ 1.8, MgCl_2_ 1, MES 5, pH 5.0 with Tris. Bath solution exchanges were controlled by a magnetic valve system (ALA BPS-8) in combination with a TIB14 interface (HEKA). Voltage clamp experiments were performed using an OC-725 C amplifier (Warner Instruments Corp.) interfaced via a LIH-1600 (HEKA) to a PC with PULSE 8.67 software (HEKA) for data acquisition and analysis. Oocytes were clamped at a holding potential of −60 mV. Downward current deflections in the current traces correspond to inward currents, i.e., movement of positive charge from the extracellular side into the cell. Amiloride-sensitive whole-cell currents (Δ*I*_ami_) were determined by washing out amiloride with amiloride-free ND96 and subtracting the whole-cell currents measured in the presence of amiloride from the corresponding whole-cell currents recorded in the absence of amiloride. Δ*I*_ami_ was determined twice in a single oocyte, i.e., before and after exposure to a test solution. To recover from the first measurement of Δ*I*_ami_, the oocyte was placed for 5 min in ND96. Subsequently, the oocyte was transferred to 150 μl of test solution (protease- and/or inhibitor-supplemented ND96 or protease-free ND96 solution as control) and was incubated for 30 min before Δ*I*_ami_ was determined for the second time.

### Detection of ENaC cleavage products at the cell surface

Biotinylation experiments were performed essentially as previously described [20, 45] using 30 oocytes per group. All biotinylation steps were performed at 4 °C. In some experiments, oocytes were pre-incubated for 30 min either in ND96 solution or in ND96 solution containing chymotrypsin (2 μg/ml), Cat-S (1 μM), or combination of Cat-S (1 μM) and LHVS (5 μM). After washing the oocytes three times with ND96 solution, they were incubated in the biotinylation buffer (in mM: triethanolamine 10, NaCl 150, CaCl_2_ 2, EZ-link sulfo-NHS-SS-Biotin (Pierce) 1 mg/ml, pH 9.5) for 15 min with gentle agitation. The biotinylation reaction was stopped by washing the oocytes twice for 5 min with quench buffer (in mM: glycine 192, Tris–Cl 25, pH 7.5). Subsequently, the oocytes were lysed by passing them through a 27-gauge needle in lysis buffer (in mM: NaCl 500, EDTA 5, Tris–Cl 50, pH 7.4) supplemented with protease inhibitor cocktail (“Complete Mini EDTA-free” protease inhibitor cocktail tablets, Roche Diagnostics) according to the manufacturer’s instructions. The lysates were centrifuged for 10 min at 1,500 × *g*. Supernatants were transferred to 1.5-ml Eppendorf tubes and incubated with 0.5 % Triton X-100 and 0.5 % Igepal CA-630 for 20 min on ice. Biotinylated proteins were precipitated with 100 μl of Immunopure immobilized Neutravidin beads (Pierce) washed with lysis buffer. After overnight incubation at 4 °C with overhead rotation, the tubes were centrifuged for 3 min at 1,500 × *g*. Supernatants were removed, and beads were washed three times with lysis buffer. One hundred microliters of 2× SDS-PAGE sample buffer (Rotiload 1, Roth) was added to the beads. Samples were boiled for 5 min at 95 °C and centrifuged for 3 min at 20,000 × *g* before loading the supernatants on a 10 % SDS-PAGE. To detect γENaC cleavage fragments, we used a subunit specific antibody against human γENaC at a dilution of 1:10,000 [20]. Horseradish peroxidase-labeled secondary goat anti-rabbit antibody (Santa Cruz Biotech) was used at a dilution of 1:50,000. Chemiluminescence signals were detected using ECL Plus (Amersham, GE Healthcare). Densitometric analysis was done with ImageJ 1.38x (National Institutes of Health).

### Cell culture

The M-1 mouse renal collecting duct cell line (ATCC 2038CRL, American Type Culture Collection, Rockville, MD, USA) was established by Dr G. Fejes Tóth [51]. Cells were used from passage 27 to 29 and were handled as described previously [4, 16, 52]. Cells were maintained in a 5 % CO_2_ atmosphere at 37 °C in PC1 culture medium (Lonza, Verviers, Belgium) supplemented with 2 mM glutamine, 100 U/ml penicillin, and 100 μg/ml streptomycin. For transepithelial studies, cells were seeded onto permeable Millicell-HA culture plate inserts (Millipore GmbH, Schwalbach, Germany). Cells were grown to confluence and equivalent short-circuit current (*I*_SC_) measurements were performed in Ussing chambers essentially as described previously [4, 52]. To minimize ENaC activation by endogenous proteases, the confluent M-1 cells grown on filters were pre-incubated for 4 to 6 h before the experiment with the broad spectrum serine protease inhibitor nafamostat mesylate/FUT-175 (Tocris, Bristol, UK) which was added to the apical bath solution. A 10-mM stock solution of nafamostat mesylate was prepared in H_2_O and stored at −20 °C. Before the experiment, the 10-mM stock was again diluted to 100 μM in 0.9 % NaCl. The final concentration of nafamostat mesylate applied to the cells was 1 μM.

### High-performance liquid chromatography (HPLC) and matrix-assisted laser desorption ionization-time of flight analysis (MALDI-TOF)

23-mer γENaC peptide (500 μM) was incubated with 1 μM Cat-S in 50 mM Tris–HCl, pH 7.4, for 30 min at 37 °C. Products were separated by reversed-phase HPLC and identified using MALDI-TOF. Mass spectrometry data were provided by the Bio-Organic Biomedical Mass Spectrometry Resource at UCSF (A.L. Burlingame, Director) supported by the Biomedical Research Technology Program of the NIH National Center for Research Resources, NIH NCRR P41RR001614 and 1S10RR014606.

### Statistical methods

Data are presented as mean ± SEM. *N* indicates the number of different batches of oocytes, and *n* the number of individual oocytes studied. Statistical significance was assessed by an appropriate version of Student’s *t* test with GraphPad Prism 5.04 (GraphPad Software) for Windows.

## Results

### Cat-S stimulates ENaC currents in *X. laevis* oocytes expressing human ENaC

**Fig. 1:**
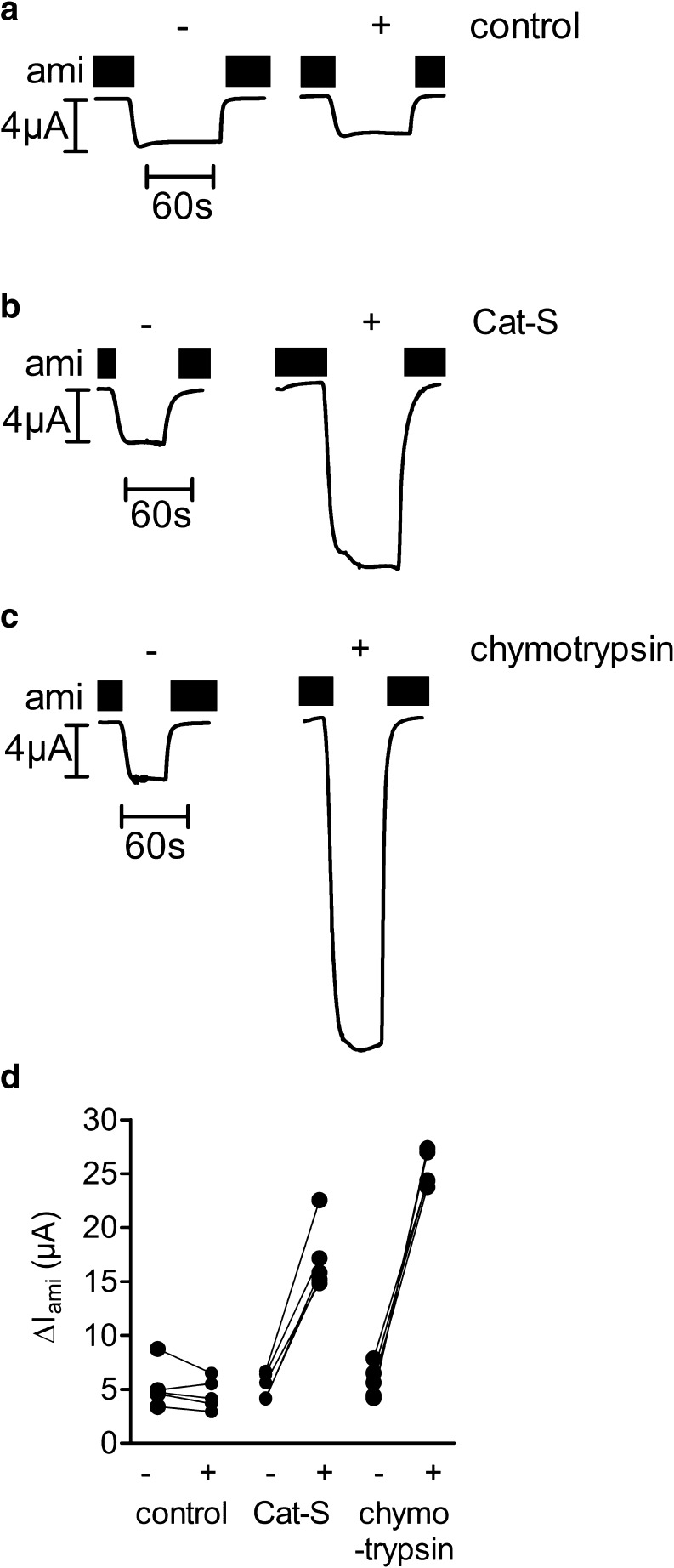
Cat-S stimulates ENaC currents in *Xenopus laevis* oocytes expressing human ENaC. **a**–**d** Oocytes expressing human ENaC were incubated for 30 min in protease-free solution (control) or in a solution containing either Cat-S (1 μM) or chymotrypsin (2 μg/ml). Amiloride-sensitive whole-cell currents (Δ*I*_ami_) were determined before (−) and after (+) incubation. Six representative whole-cell current traces from one batch of oocytes are shown. **a**–**c** Amiloride (ami) was present in the bath solution to specifically inhibit ENaC as indicated by *black bars*. **d** Individual Δ*I*_ami_ values from a representative experiment using one batch of oocytes. *Data points* obtained from an individual oocyte are connected by a *line*

With the exception of meprin β, only serine proteases have been shown to activate ENaC. Using the two-electrode voltage clamp technique, we investigated whether the cysteine protease Cat-S can also activate ENaC. We determined amiloride-sensitive whole-cell currents (Δ*I*_ami_) of individual ENaC-expressing oocytes before and after 30 min of incubation of the oocytes in Cat-S, chymotrypsin, or protease-free solution. Chymotrypsin (2 μg/ml) is a prototypical serine protease known to elicit a near maximal stimulatory effect on ENaC [12]. Figure 1a–c shows six representative whole-cell current traces from one batch of oocytes. Each individual oocyte was measured twice, i.e., before and after a 30-min exposure to protease-free solution (Fig. 1a), to Cat-S (Fig. 1b), or to chymotrypsin (Fig. 1c) solution. In Fig. 1d, the initial Δ*I*_ami_ values measured in one batch of oocytes were connected by lines to the corresponding values measured after 30 min. Exposure to Cat-S or chymotrypsin increased Δ*I*_ami_ in each oocyte measured. In contrast, in control experiments, a 30-min incubation of ENaC-expressing oocytes in protease-free solution had a negligible effect on ENaC currents (Fig. 1d). In conclusion, we demonstrated that Cat-S can activate ENaC currents in αβγ-human ENaC-expressing oocytes.

### Stimulatory effect of Cat-S on human ENaC is concentration dependent

**Fig. 2:**
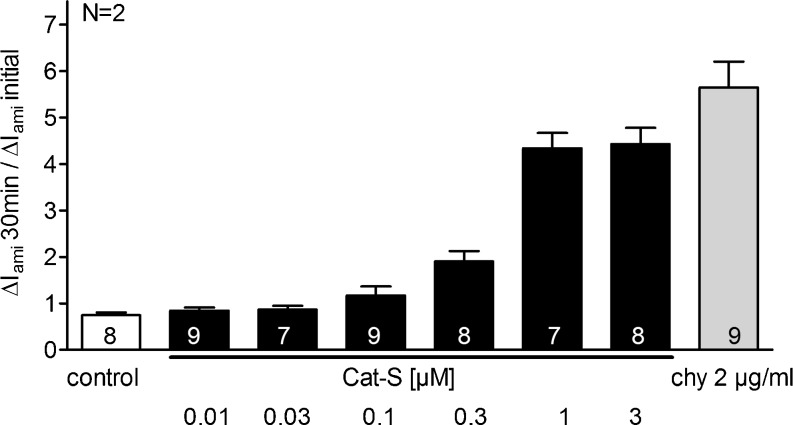
Stimulatory effect of Cat-S on human ENaC is concentration dependent. Oocytes expressing human αβγ ENaC were incubated for 30 min in protease-free solution (control), in solutions containing different concentrations of Cat-S (0.01, 0.03, 0.1, 0.3, 1, and 3 μM) or in a solution containing chymotrypsin (2 μg/ml). Amiloride-sensitive whole-cell currents (Δ*I*_ami_) were detected before (Δ*I*_ami_ initial) and after incubation (Δ*I*_ami_ 30 min). *Columns* represent the relative stimulatory effect on Δ*I*_ami_ calculated as the ratio of Δ*I*_ami_ 30 min/Δ*I*_ami_ initial. *Numbers inside the columns* indicate the number of individual oocytes measured. *N* indicates the number of different batches of oocytes

To investigate the concentration dependence of the Cat-S effect, we performed experiments using different concentrations of Cat-S (0.01, 0.03, 0.1, 0.3, 1, and 3 μM) (Fig. 2). As expected, Cat-S increased Δ*I*_ami_ in a concentration-dependent manner. The effect of 3 μM Cat-S was not larger than that of 1 μM Cat-S which was the concentration routinely used in our oocyte experiments. With the Cat-S preparations available for the present study, we could not further increase the Cat-S concentration.

### Activation of ENaC by Cat-S is prevented by the Cat-S inhibitor LHVS

To confirm that the observed ENaC activation is caused by the Cat-S activity of the protease preparation used and not by contamination with a serine protease, we examined the effect of an irreversible Cat-S inhibitor (LHVS) on proteolytic ENaC activation by recombinant Cat-S. Peptidyl vinyl sulfones are specific cysteine protease inhibitors of human cathepsins [6]. The active site cysteine of the cysteine protease Cat-S covalently binds to the vinylsulfone residue. This reaction with the target cysteine protease is irreversible.

**Fig. 3:**
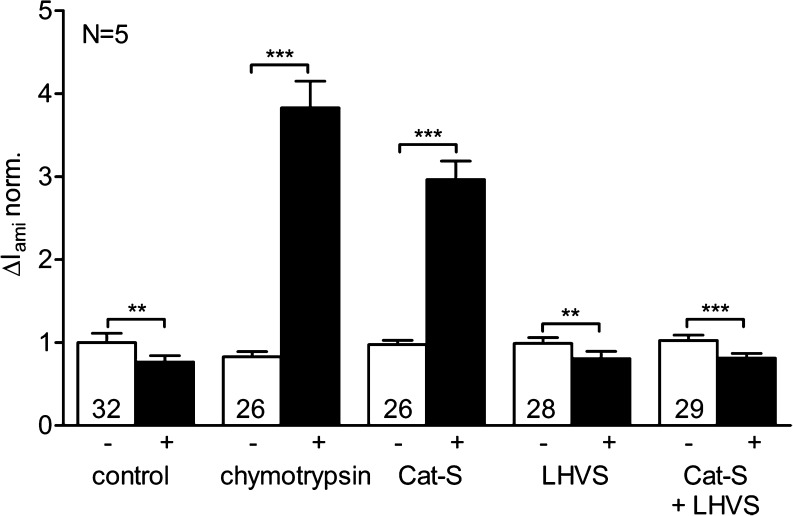
Activation of ENaC by Cat-S is prevented by the Cat-S inhibitor LHVS. Oocytes expressing human ENaC were incubated for 30 min in protease-free solution (control), in chymotrypsin (2 μg/ml), in Cat-S (1 μM), in LHVS (2 μM), or in a solution containing a combination of Cat-S (1 μM) and LHVS (5 μM). Amiloride-sensitive whole-cell currents (Δ*I*_ami_) were determined before (−) and after (+) incubation. The *bar diagram* represents normalized average results obtained in five different batches of oocytes (*N* = 5). The individual Δ*I*_ami_ values were normalized to the mean Δ*I*_ami_ value of the ENaC-expressing control group in protease-free solution. *Numbers inside the columns* indicate the number of individual oocytes measured. **p < 0.01, ***p < 0.001, paired *t* test

Δ*I*_ami_ was measured before and after 30 min of incubation of the oocytes in protease-free solution, in chymotrypsin (2 μg/ml), in Cat-S (1 μM), in LHVS (2 μM), or in a solution containing a combination of Cat-S (1 μM) and LHVS (5 μM) (Fig. 3). Chymotrypsin and Cat-S had an average stimulatory effect of about 5.3- and 3.2-fold, respectively. To test whether ENaC activation by Cat-S is prevented by the Cat-S inhibitor LHVS, the solution containing Cat-S (1 μM) was pre-incubated with the inhibitor (5 μM) for 10 min. Subsequently, oocytes were incubated for 30 min in this Cat-S solution containing LHVS before ENaC currents were measured again. LHVS completely prevented the stimulation of ENaC currents by recombinant Cat-S used in our experiments. Incubation of oocytes in LHVS alone slightly reduced ENaC currents which probably can be attributed to the well-known phenomenon of channel “rundown” [53] also observed in control experiments with protease-free solution. The finding that LHVS prevents ENaC stimulation by the Cat-S preparation used indicates that the stimulatory effect is mediated by Cat-S.

### The Cat-S inhibitor LHVS has no effect on ENaC activation by the serine proteases chymotrypsin and trypsin

**Fig. 4:**
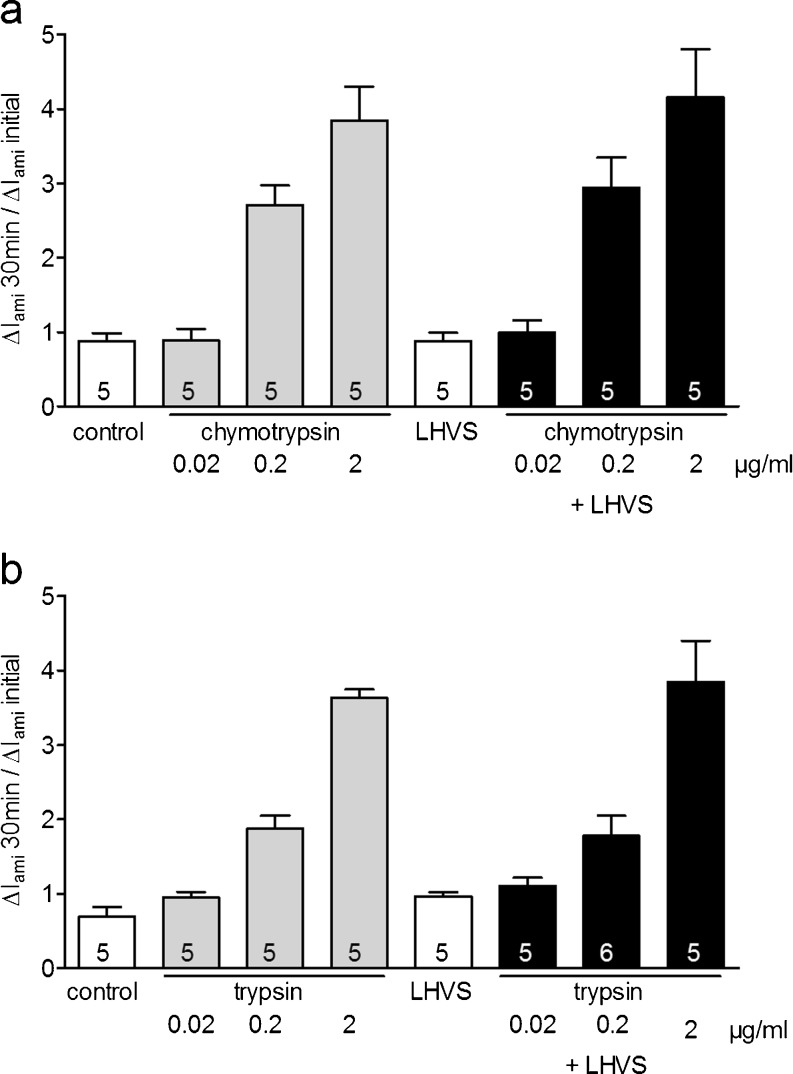
The Cat-S inhibitor LHVS has no effect on ENaC activation by the serine proteases trypsin and chymotrypsin. **a** Oocytes expressing human ENaC were incubated for 30 min in protease-free solution (control), in solutions with different concentrations of chymotrypsin (0.02, 0.2, and 2 μg/ml), in LHVS (2 μM), or in solutions containing a combination of different concentrations of chymotrypsin (0.02, 0.2, and 2 μg/ml) and LHVS (2 μM). Amiloride-sensitive whole-cell currents (Δ*I*_ami_) were determined before (−) and after (+) incubation. *Columns* represent relative stimulatory effect on Δ*I*_ami_ calculated as the ratio of Δ*I*_ami_ measured after 30 min of incubation (Δ*I*_ami_ 30 min) to the initial Δ*I*_ami_ (Δ*I*_ami_ initial) measured before incubation. *Numbers inside the columns* indicate the number of individual oocytes measured. **b** Similar experiment as shown in **a** using the serine protease trypsin (2 μg/ml) instead of chymotrypsin

To rule out the possibility that the Cat-S inhibitor LHVS may have a nonspecific inhibitory effect on serine proteases, we also tested the effect of LHVS on ENaC activation by the prototypical serine proteases chymotrypsin and trypsin (Fig. 4a, b). We measured Δ*I*_ami_ before and after incubation of the oocytes for 30 min in a protease-free solution, in solutions with different concentrations of chymotrypsin (0.02, 0.2, 2 μg/ml), in LHVS (2 μM), or in solutions containing a combination of different concentrations of chymotrypsin and LHVS (2 μM) (Fig. 4a). As expected, exposure to different concentrations of chymotrypsin increased Δ*I*_ami_ in a concentration-dependent manner. To investigate the effect of the Cat-S inhibitor LHVS on ENaC activation by the serine protease chymotrypsin, solutions containing different concentrations of chymotrypsin were pre-incubated with LHVS (2 μM) for 10 min. Afterwards, the oocytes were incubated in these chymotrypsin solutions containing LHVS and Δ*I*_ami_ was determined. In contrast to the inhibition of Cat-S by administration of LHVS (see Fig. 3), LHVS had no significant effect on the activation of ENaC by different concentrations of chymotrypsin. Similar results were obtained using the serine protease trypsin (Fig. 4b). In summary, these findings indicate that in the concentration used, LHVS has no inhibitory effect on the serine proteases chymotrypsin and trypsin. This demonstrates that the inhibitory effect of LHVS on ENaC activation by Cat-S is specific and not caused by contamination with serine proteases.

### Cat-S can activate ENaC in an acidic environment

**Fig. 5:**
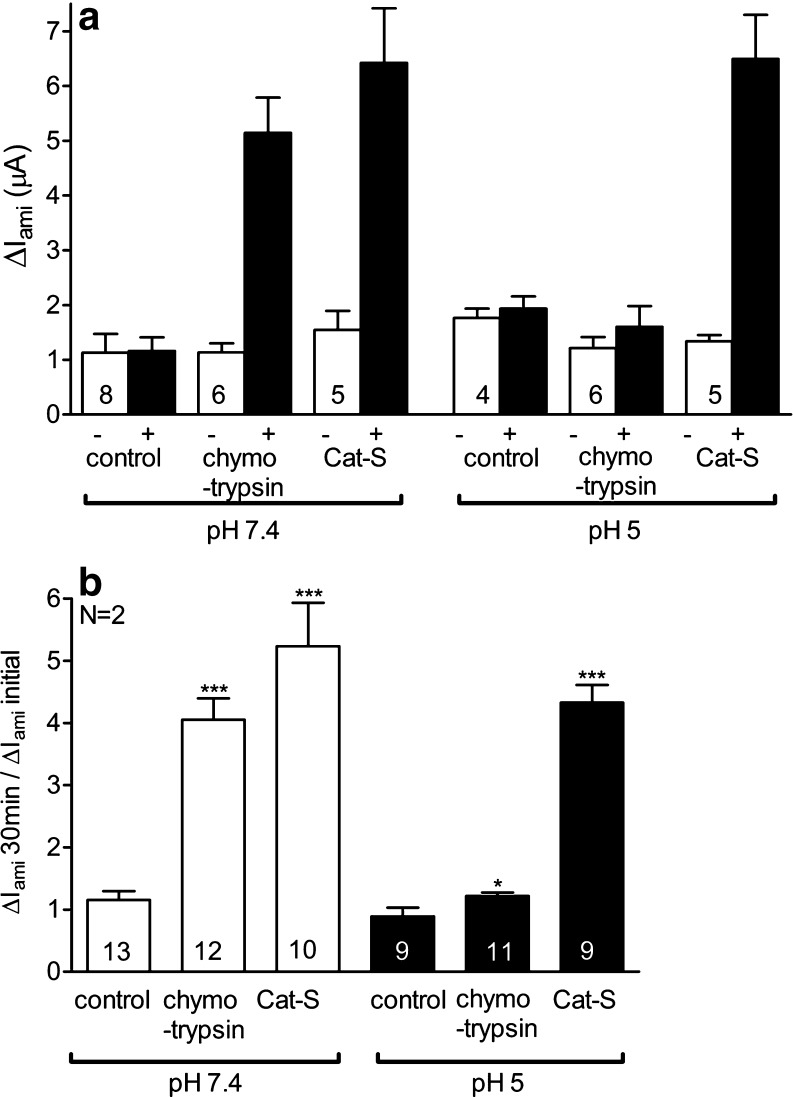
Cat-S can activate ENaC in an acidic environment. Oocytes expressing human ENaC were incubated for 30 min in protease-free (control), in chymotrypsin (0.2 μg/ml), or in Cat-S (1 μM) solution with a physiological pH of 7.4 (*white columns*) or an acidic pH of 5 (*black columns*). Amiloride-sensitive whole-cell currents (Δ*I*_ami_) were determined before (−) and after (+) incubation. **a** Δ*I*_ami_ values from a representative experiment using one batch of oocytes. **b** Summary of similar experiments as shown in **a**. *Columns* represent relative stimulatory effect on Δ*I*_ami_ calculated as the ratio of Δ*I*_ami_ measured after 30 min of incubation (Δ*I*_ami_ 30 min) to the initial Δ*I*_ami_ (Δ*I*_ami_ initial) measured before incubation. *Numbers inside the columns* indicate the number of individual oocytes measured. *N* indicates the number of different batches of oocytes. *p < 0.05, ***p < 0.001, unpaired *t* test

Inflammation is often associated with tissue acidification (pH values from 5 to 6) and the activity of proteases is known to be pH sensitive. To test whether Cat-S can also activate ENaC in an acidic environment, we performed similar experiments as described in Fig. 1 and exposed matched groups of oocytes either to pH 7.4 or to pH 5 (Fig. 5). Extracellular pH has been reported to affect ENaC activity by complex mechanisms [13, 14]. However, in the experiments summarized in Fig. 5a, baseline Δ*I*_ami_ values of ENaC-expressing oocytes were not significantly different in the two groups of oocytes. In this set of experiments, the stimulatory effect of Cat-S on ENaC was about 5.2-fold at pH 7.4 (Fig. 5b). Importantly, this stimulatory effect of Cat-S on ENaC activity was largely preserved at pH 5 (4.3-fold). In contrast, the stimulatory effect of chymotrypsin (0.2 μg/ml) was almost completely suppressed at pH 5. Our data indicate that Cat-S can activate ENaC in an acidic environment.

### In vitro cleavage analysis of the 23-mer γENaC peptide suggests that Cat-S may cleave human γENaC

**Fig. 6:**
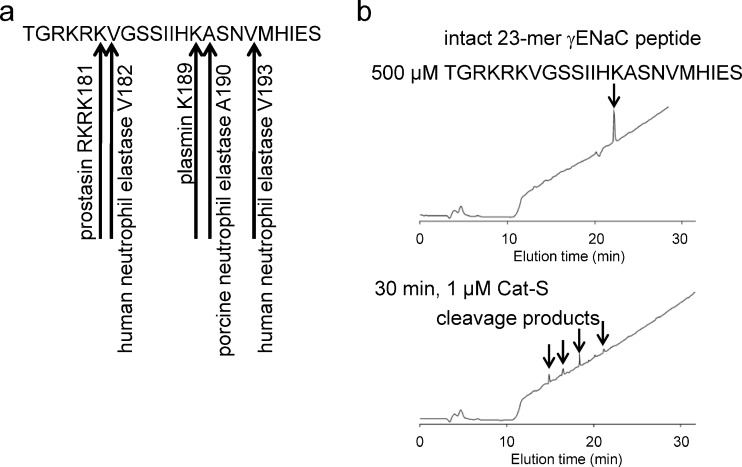
In vitro cleavage analysis of the 23-mer γENaC peptide suggests that Cat-S may cleave human γENaC at more than one cleavage site. **a** Sequence of the 23-mer γENaC peptide showing putative cleavage sites for proteolytic ENaC activation. **b** The 23-mer γENaC peptide (500 μM) was incubated with Cat-S (1 μM) for 30 min and cleavage products were identified by HPLC and mass spectrometry. Cat-S degraded the 23-mer γENaC peptide showing four products detected by HPLC

To identify a putative cleavage site(s) for Cat-S, a 23-mer γENaC peptide (176-TGRKRKVGGSIIHKASNVMHIES-198) was synthesized that corresponds to a region in the extracellular domain of γENaC thought to contain cleavage sites critical for proteolytic channel activation [1] (Fig. 6a). The 23-mer γENaC peptide was incubated with Cat-S (1 μM) for 30 min and proteolytic degradation was assessed using HPLC and MALDI-TOF mass spectrometry (Fig. 6b). As evidenced by the appearance of several peaks, Cat-S was able to cleave the 23-mer γENaC peptide at more than one cleavage site.

### Activation of ENaC by Cat-S generates a γENaC cleavage product at the cell surface indicating proteolytic channel activation

Proteolytic activation of ENaC is associated with the appearance of different cleavage products. In addition to a 87-kDa band for full-length γENaC, a cleavage product of about 76 kDa appears when γENaC is co-expressed with α- and βENaC [7, 17, 22, 23, 26, 52]. This 76-kDa cleavage product results from cleavage of γENaC by endogenous proteases like the Golgi-associated convertase furin at the so-called furin cleavage site. An additional 67-kDa band can be detected following a second cleavage event in a region in γENaC distal to the furin site. This second and functionally relevant final cleavage step of proteolytic ENaC stimulation is critical for the activation of membrane resident near-silent channels [17] and is usually mediated by membrane-bound or extracellular proteases, e.g., by plasmin, chymotrypsin, or trypsin.

**Fig. 7:**
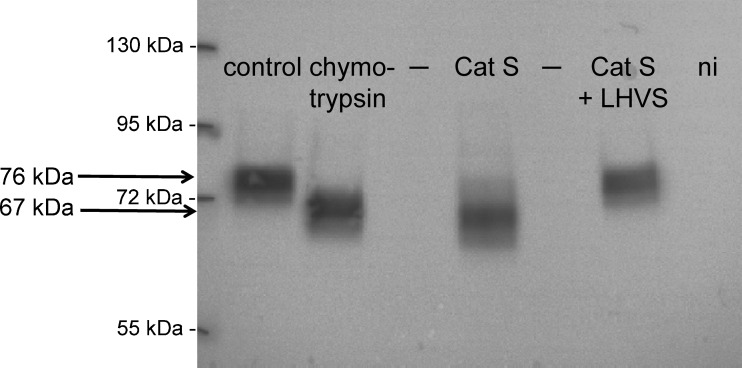
Activation of ENaC by Cat-S generates a γENaC cleavage product at the cell surface indicating proteolytic channel activation. The Cat-S inhibitor LHVS prevented the generation of an additional cleavage product at the cell surface. Oocytes expressing human ENaC were incubated for 30 min in protease-free solution (control), in chymotrypsin (2 μg/ml), in Cat-S (1 μM), or in a solution containing a combination of Cat-S (1 μM) and LHVS (5 μM). Expression of biotinylated γENaC at the cell surface was analyzed by SDS-PAGE. γENaC was detected with an antibody against the C-terminus of human γENaC. In non-injected (ni) oocytes, γENaC-specific signals were absent. “–” indicates an empty lane on the gel. Molecular weight markers are shown on the *left side* of the gel. Representative western blot from one batch of oocytes

Using a biotinylation approach as previously described [20, 45], we investigated whether ENaC activation by Cat-S also results in the appearance of this 67-kDa γENaC fragment at the cell surface. For this purpose, ENaC-expressing oocytes were treated for 30 min with protease-free solution, chymotrypsin (2 μg/ml), or Cat-S (1 μM) solution. Subsequently, the biotinylated γENaC cleavage products were detected by western blot using a γENaC antibody directed against an epitope at the C-terminus (Fig. 7). The predominant γENaC fragment detected at the cell surface of untreated ENaC-expressing control oocytes had a molecular weight of about 76 kDa. The signal for full-length γENaC (87 kDa) usually was not detectable which is in agreement with previously reported data [17, 22, 23, 52]. As expected, activation of ENaC by exposure to chymotrypsin resulted in the disappearance of the 76-kDa band and the appearance of a lower size cleavage fragment with a molecular weight of about 67 kDa. Interestingly, incubation of the oocytes in Cat-S solution had a similar effect causing the 76-kDa band to disappear and producing a cleavage product that appeared slightly smaller than 67 kDa. These results indicate that Cat-S also causes cleavage of the γ-subunit distal to the furin cleavage site and possibly slightly more distal than chymotrypsin.

As the stimulatory effect of Cat-S on ENaC currents was prevented by the Cat-S inhibitor LHVS, we investigated whether LHVS also prevented proteolytic cleavage of γENaC. Therefore, the solution containing Cat-S (1 μM) was pre-incubated with the inhibitor (5 μM) for 10 min. Subsequently, oocytes were incubated for 30 min in this Cat-S solution containing LHVS before cell surface expressed γENaC cleavage products were investigated. As shown in Fig. 7, the Cat-S inhibitor LHVS prevented the generation of the 67-kDa γENaC cleavage product at the cell surface. Thus, our findings are consistent with the result that the inhibitor prevented proteolytic activation of ENaC currents by Cat-S.

### Mutating two putative neutrophil elastase cleavage sites (γ_V182;V193_) prevents proteolytic activation of γENaC by Cat-S

**Fig. 8:**
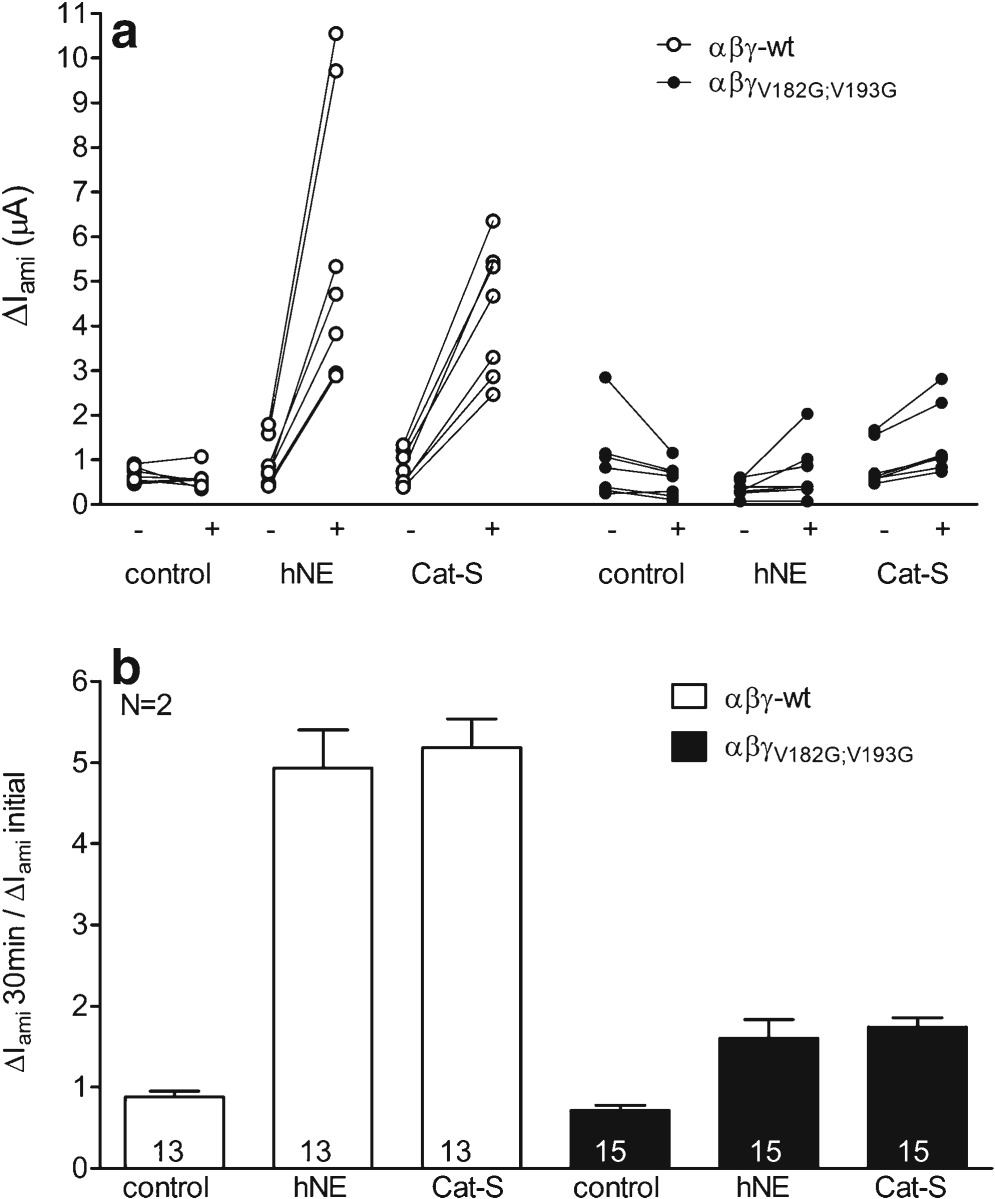
Mutating two putative neutrophil elastase cleavage sites (γV182;V193) prevents proteolytic activation of γENaC by Cat-S. Oocytes expressing αβγ (*open symbols*) or αβγ_V182G;V193G_ENaC (*filled symbols*) were incubated for 30 min in protease-free solution (control) or in a solution containing either hNE (10 μg/ml) or Cat-S (1 μM). Amiloride-sensitive whole-cell currents (Δ*I*_ami_) were determined before (−) and after (+) incubation. **a** Individual Δ*I*_ami_ values from a representative experiment using one batch of oocytes. *Data points* obtained from individual oocytes are connected by a *line*. **b** Summary of similar experiments as shown in **a**. *Columns* represent relative stimulatory effect on Δ*I*_ami_ calculated as the ratio of Δ*I*_ami_ measured after 30 min of incubation (Δ*I*_ami_ 30 min) to the initial Δ*I*_ami_ (Δ*I*_ami_ initial) measured before incubation. *Numbers inside the columns* indicate the number of individual oocytes measured. *N* indicates the number of different batches of oocytes

Cat-S is known to preferentially target the amino acids leucine or valine. Interestingly, there are two valine residues (V182 and V193) located in the region of γENaC where the final cleavage event is thought to occur that leads to channel activation. These cleavage sites previously have been described as cleavage sites for hNE [1]. To investigate the functional relevance of these sites for channel activation by Cat-S, we generated a γENaC construct with a double mutation γ_V182G;V193G_ and tested the effect of hNE or Cat-S on ENaC currents of oocytes expressing wt αβγENaC or mutant αβγ_V182G;V193G_ENaC. We measured Δ*I*_ami_ in individual oocytes before and after 30 min of exposure to protease-free solution, hNE (10 μg/ml), or Cat-S (1 μM). Baseline Δ*I*_ami_ values of wt and αβγ_V182G;V193G_ mutant expressing oocytes were of similar size (Fig. 8a). As shown in Fig. 8b, hNE stimulated Δ*I*_ami_ of wt ENaC-expressing oocytes to a similar extent as Cat-S. Mutating the relevant hNE cleavage sites should diminish or prevent activation of the mutant channel by hNE. Indeed, the stimulatory effect of hNE was almost completely abolished in αβγ_V182G;V193G_ENaC. Interestingly, the γ_V182G;V193G_ mutation also prevented proteolytic activation of ENaC by Cat-S. In control experiments, a 30-min incubation of wt and mutant ENaC-expressing oocytes in protease-free solution had a negligible effect on ENaC currents. In conclusion, we have demonstrated that mutating the two valine residues (V182 and V193) prevents the stimulatory effect of Cat-S on ENaC activation. These data suggest that the putative cleavage sites V182 and V193 for hNE are also likely cleavage sites for Cat-S.

### Cat-S stimulates ENaC in confluent M-1 mouse collecting duct cells

**Fig. 9:**
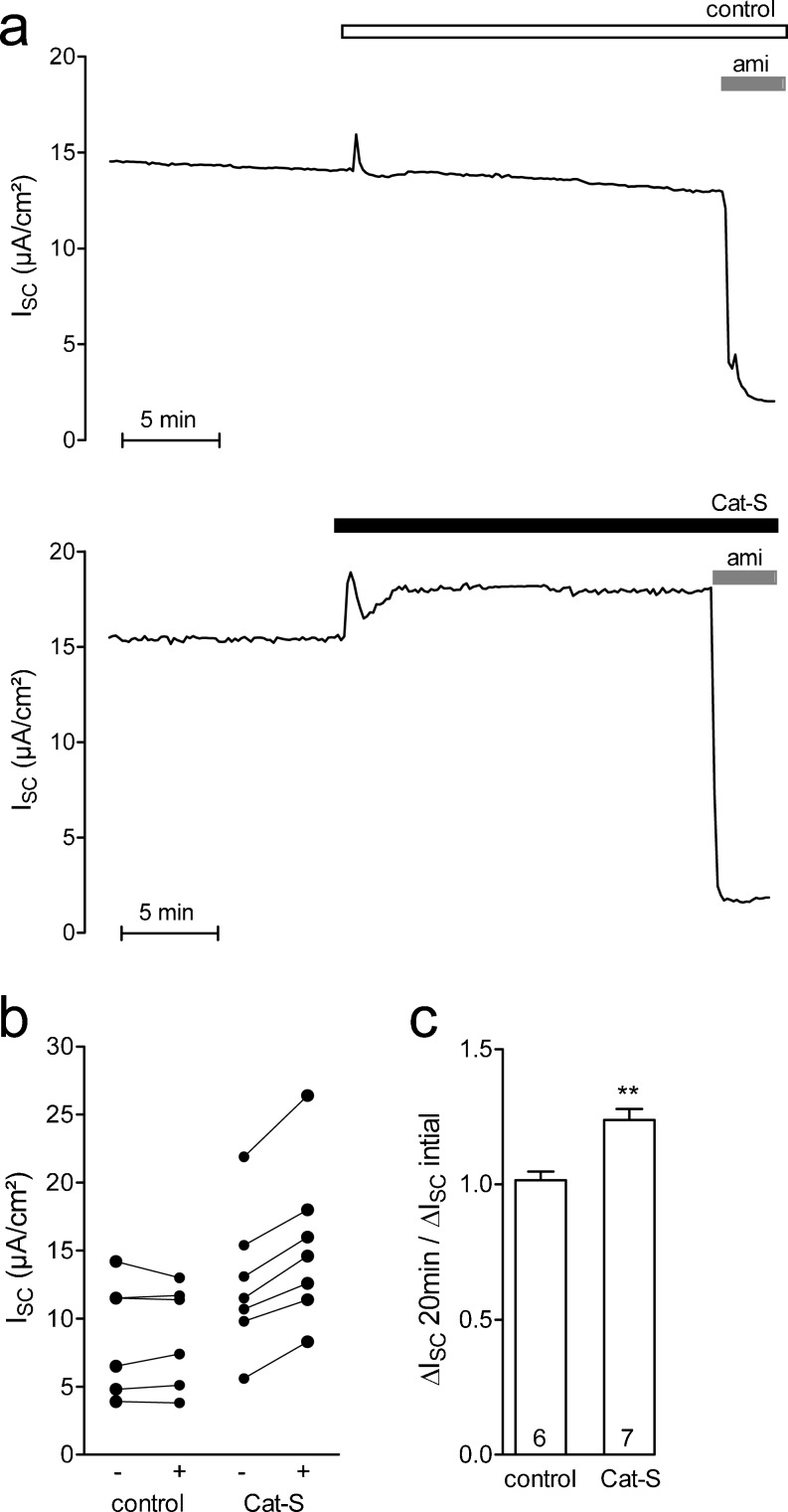
Cat-S stimulates ENaC in confluent M-1 mouse collecting duct cells. **a** Representative equivalent short-circuit current (*I*_SC_) recordings from confluent M-1 cells pretreated with nafamostate mesylate to reduce constitutive ENaC activation by endogenous proteases. Vehicle control (phosphate buffered saline, *upper trace*) or Cat-S (2 μM, *lower trace*) was added to the apical bath solution of M-1 cells. At the end of the experiment, amiloride (ami; 10 μM) was added apically to confirm that the stimulated *I*_SC_ was mediated by ENaC. **b**, **c** Summary of results from similar experiments as shown in **a**. **b** *Data points* represent individual *I*_SC_ values obtained from six (control) or seven (Cat-S) individual experiments that are connected by a *line*. **c** *Columns* represent relative stimulatory effect on Δ*I*_SC_ calculated as the ratio of Δ*I*_SC_ measured after 20 min of incubation (Δ*I*_SC_ 20 min) to the initial *I*_SC_ (Δ*I*_SC_ initial) measured before apical addition. **p < 0.01, unpaired *t* test

In addition to the effect of Cat-S in the oocyte system, we investigated the effect of Cat-S in cultured M-1 mouse collecting duct cells known to express ENaC when grown to confluence in PC1 culture medium containing a high concentration (5 μM) of dexamethasone [4, 52]. Figure 9a shows two representative short-circuit current traces. It is well established that in M-1 cells under the experimental conditions used, baseline *I*_SC_ can be attributed to ENaC-mediated electrogenic sodium transport [4, 16, 24]. In the upper control trace, baseline *I*_SC_ shows a typical slow decline over time which is probably caused by spontaneous channel rundown. Application of vehicle (phosphate buffered saline) resulted in a transient solution exchange artifact but did not affect baseline *I*_SC_. Application of amiloride (10 μM) largely inhibited *I*_SC_ which confirmed that it was mediated by ENaC. In the lower trace, the initial *I*_SC_ is similar to that in the control trace. Importantly, apical application of Cat-S (2 μM) resulted in a sustained increase in *I*_SC_ which remained sensitive to amiloride. This indicates that the *I*_SC_ increase observed upon application of Cat-S is caused by a stimulation of ENaC. The stimulatory response to Cat-S was observed in all experiments (*n* = 7) in which Cat-S was applied (Fig. 9b). On average, apical application of Cat-S (2 μM) stimulated ENaC-mediated *I*_SC_ by about 24 % (Fig. 9c).

## Discussion

In the present study, we showed that Cat-S stimulates ENaC-mediated whole-cell currents in *X. laevis* oocytes expressing human ENaC. This stimulation was associated with the appearance of a γENaC cleavage fragment at the cell surface indicating proteolytic channel activation. The stimulatory effect of Cat-S on ENaC activity and the concomitant appearance of a γENaC cleavage product at the cell surface were prevented by the Cat-S inhibitor LHVS. In addition, we demonstrated that Cat-S can stimulate ENaC-mediated transepithelial sodium transport in differentiated renal epithelial cells. To our knowledge, this is the first report that the cysteine protease Cat-S activates ENaC most likely by proteolytic cleavage of its γ-subunit at the cell surface. Stimulation of ENaC by locally released Cat-S may play a pathophysiological role in inflammatory disease, e.g., in colitis or nephritis.

As a rate-limiting step for transepithelial Na^+^ transport, ENaC is the main target of highly complex regulatory mechanisms that adjust transepithelial Na^+^ transport to levels appropriate for tissue needs and for overall body sodium homeostasis. In addition to extracellular and intracellular proteases, these mechanisms include hormones (e.g., aldosterone, angiotensin II, vasopressin, insulin, insulin-like growth factor I), intra- and extracellular ion concentrations, osmolarity, tubular flow rate, kinases (e.g., serum- and glucocorticoid-inducible kinase isoform 1, protein kinase A, extracellular-regulated kinase), and interacting proteins (e.g., ubiquitin ligases, deubiquitinylating proteases, Rab proteins) [35]. At present, it is unclear how these mechanisms interact and how for example the hormonal regulation of ENaC is linked to its proteolytic activation. In the kidney, aldosterone is thought to be the main hormonal regulator of ENaC activity. Interestingly, high levels of aldosterone known to activate ENaC activity in the distal tubule have also been shown to increase proteolytic ENaC cleavage [18, 33, 36]. This stimulatory effect on ENaC cleavage may be mediated by an aldosterone-induced expression of prostasin [38]. This example of a possible link between the hormonal and proteolytic regulation of the channel makes it likely that other physiological pathways of ENaC regulation also involve proteases which may include different types of proteases in a tissue-specific manner.

Most of our knowledge about proteolytic ENaC activation stems from studies in model systems like *X. laevis* oocytes and cultured cells. Recently, functional evidence is emerging that ENaC activation by extracellular proteases also occurs in native tissue. Indeed, it has been demonstrated that trypsin can activate ENaC in microdissected mouse [39] and rat [18] distal nephron. Moreover, in kallikrein-deficient mice, the natriuretic effect of amiloride and the amiloride-sensitive rectal potential difference were found to be reduced compared to wild-type animals [43]. These results suggested that kallikrein can activate ENaC which was confirmed in a recent study using the oocyte expression system [42].

Interestingly, the stimulatory effect of Cat-S on ENaC currents was more pronounced in the oocyte expression system heterologously expressing ENaC than in the M-1 mouse collecting duct cells endogenously expressing ENaC. This discrepancy is consistent with previously reported findings that baseline ENaC activity in M-1 cells is high which may result from the culture conditions used. Indeed, to reveal proteolytic ENaC activation by soluble proteases applied to the apical surface of M-1 cells, the cells need to be pretreated with inhibitors of endogenous proteases. However, even in pretreated cells, the proteolytic activation of ENaC by trypsin or plasmin was found to be smaller in M-1 cells than that observed in the oocyte expression system [34, 39, 52]. These findings are consistent with those of the present study and suggest that constitutive proteolytic activation of ENaC by endogenous proteases is more complete in M-1 cells than in the oocyte expression system.

There is convincing evidence that proteolytic processing by serine proteases in the biosynthetic pathway and at the plasma membrane is essential for the regulation of ENaC activity [32, 47]. However, to our knowledge so far, no data have been reported demonstrating an effect of cathepsin proteases or of other members of the group of cysteine proteases on ENaC activity. Cysteine proteases are secreted by inflammatory and epithelial cells during injury and disease. Cat-S is expressed in the kidney, spleen, lymph nodes, and lung [31] and also by antigen-presenting cells and macrophages which allow its secretion in a wide range of different tissues and organs [11, 57]. Cat-S levels in the cerebrospinal fluid have been reported to be in the low nanomolar range [40]. In contrast, in lysosomes, there are some reports demonstrating millimolar levels of cathepsins [8, 55]. In the kidney, the strongest expression of Cat-S was detected in proximal tubule cells [31]. During inflammatory diseases, Cat-S may be secreted by proximal tubule cells into the urine and hence may reach ENaC in the distal tubule. Recent data show that Cat-S is selectively activated in the colonic lumen during colitis [11]. Thus, in the colonic lumen, Cat-S may also reach epithelial cells expressing ENaC at the luminal membrane. However, the concentration of Cat-S that may be reached at the apical membrane of epithelial cells in inflammatory disease is not yet known.

We showed that the stimulation of ENaC by Cat-S and chymotrypsin generates a ~67-kDa γENaC cleavage product at the cell surface. Previously, a similar 67-kDa γENaC cleavage fragment has been described after ENaC activation by trypsin [17], plasmin [41], neutrophil elastase [22], or by co-expression of ENaC with prostasin [7]. This suggests that the cleavage sites in γENaC mediating proteolytic channel activation by all these proteases including Cat-S are localized in close vicinity. Putative cleavage sites for prostasin (CAP1) (γRKRK178, [7]), plasmin (γK189, [41]), and neutrophil elastase (γV182;γV193, [1]) have been described and are located distal to the furin cleavage site in the extracellular domain of the γ-subunit. According to the availability of proteases in tissues, different protease cleavage sites may be used in distinct tissues to cleave and activate ENaC.

Physiologically relevant protease cleavage sites are difficult to predict from the amino acid sequence of a protein since conformational aspects need to be considered. Cat-S is known to preferentially target the amino acids leucine or valine. There are two valine residues in the critical region in γENaC. Thus, it is possible that Cat-S cleaves in this region. Our mass spectrometry data suggest that Cat-S may cleave ENaC at more than one cleavage site in a peptide corresponding to this critical region of γENaC. Indeed, mutating the two putative cleavage sites for human neutrophil elastase (V182;V193) largely abolished the stimulatory effect of Cat-S on ENaC. Thus, the two valine residues are essential for Cat-S activation of ENaC and these are likely cleavage sites for Cat-S.

In addition, we have to consider the possibility that ENaC stimulation by Cat-S is mediated indirectly by its activating effect on other proteases, e.g., a membrane-bound protease like prostasin, which in turn proteolytically activate ENaC. However, ENaC stimulation by Cat-S was preserved at pH 5. This argues in favor of a direct proteolytic effect of Cat-S on ENaC, because most serine proteases, including those present at the plasma membrane of the oocytes, are likely to be inactive at an acidic pH of 5. Indeed, serine proteases usually are active at pH 7 to 9, whereas cysteine proteases prefer a pH range from 3 to 7. Thus, Cat-S is a protease that can proteolytically activate ENaC at acidic pH typical for inflamed tissue where serine proteases would no longer be functional. In summary, we demonstrate for the first time that ENaC can be activated by Cat-S which may be pathophysiologically relevant in inflammatory disease. This process is mediated by the neutrophil elastase cleavage sites which therefore play a key role in proteolytic ENaC activation by Cat-S.

## Abbreviations

ENaC: Epithelial sodium channel
Cat-S: Cathepsin-S
hNE: Human neutrophil elastase
LHVS: Morpholinurea-leucine-homophenylalanine-vinylsulfone-phenyl
